# COP1 drives renal cell carcinoma progression by targeting ACSL4 for ubiquitin-mediated degradation and inhibiting ferroptosis

**DOI:** 10.3389/fonc.2025.1570727

**Published:** 2025-05-06

**Authors:** Yuxiao Zheng, Lei Jiang, Feng Qi, Bo Peng

**Affiliations:** ^1^ Department of Urologic Surgery, Jiangsu Cancer Hospital and Jiangsu Institute of Cancer Research and Affiliated Cancer Hospital of Nanjing Medical University, Nanjing, China; ^2^ Department of Urologic Surgery, Zhenjiang Hospital of Chinese Traditional and Western Medicine, Zhenjiang, China; ^3^ Clinical Medical College of Shanghai Tenth People’s Hospital, Nanjing Medical University, Shanghai, China

**Keywords:** COP1, ACSL4, ubiquitination, ferroptosis, RCC

## Abstract

**Background:**

Renal cell carcinoma (RCC) progression is closely linked to dysregulation of the ubiquitin-proteasome system, particularly aberrant ubiquitination processes governing protein degradation and cell cycle control. As a pivotal E3 ubiquitin ligase, COP1 mediates substrate-specific ubiquitination to regulate protein stability. However, its functional role in RCC remains poorly characterized. This study investigates how COP1 drives RCC malignancy and explores its underlying molecular mechanisms.

**Methods:**

We analyzed the expression of COP1 in RCC cells and its relationship with patient overall survival (OS) in databases. The CCK-8 assay was used to detect the effect of COP1 on the proliferation of RCC cells, while the Transwell assay was used to assess the impact of COP1 on the migration and invasion of RCC cells. We employed mass spectrometry, co-immunoprecipitation, Western blot, and RT-qPCR to explore the target proteins that interact with COP1 and their interaction modes. After inducing with ferroptosis inducers, we measured the effect of COP1 on lipid ROS levels in RCC cells. Finally, we validated the role of COP1 in RCC using *in vivo* experiments.

**Results:**

COP1 was significantly correlated with poor patient prognosis. Functional studies demonstrated that COP1 overexpression markedly increased RCC cell proliferation by 65% (786-O) and 58% (ACHN) (*p <* 0.001) and enhanced migration/invasion (*p <* 0.01), while COP1 knockdown suppressed these malignant phenotypes by 40–50%. Mechanistically, COP1 directly bound ACSL4 and promoted its K48-linked ubiquitination, reducing ACSL4 protein stability by 70% (*p <* 0.001) and suppressing ferroptosis, as evidenced by decreased lipid ROS levels (*p <* 0.01) and reversal of ferroptosis inhibition by ferrostatin-1. *In vivo*, COP1 overexpression accelerated tumor growth in xenograft models, with a 2.5-fold increase in tumor volume compared to controls (*p <* 0.001), accompanied by reduced ACSL4 expression and elevated Ki67 proliferation index. These effects were further amplified by the ferroptosis inhibitor ferrostatin-1, underscoring COP1’s role in driving tumor progression through ferroptosis suppression.

**Conclusion:**

Our study establishes COP1 as a critical driver of RCC progression by suppressing ferroptosis through ubiquitin-mediated degradation of ACSL4, thereby providing a novel theoretical foundation for targeted therapeutic strategies in RCC.

## Introduction

Renal cell carcinoma (RCC) is one of the most common malignant tumors in the urinary system, with an annual incidence rate increase of 1.5% in the United States (1). Due to its resistance to radiotherapy and chemotherapy, as well as its occult early clinical manifestations, more than one-third of patients develop metastasis, and one-quarter of patients experience recurrence. These patients with advanced disease have a poor prognosis (2). These challenges underscore the urgency to delineate molecular drivers of RCC progression.

Ubiquitination, a post-translational modification orchestrated by E1-activating, E2-conjugating, and E3 ligase enzymes, dynamically regulates protein stability, localization, and activity, thereby modulating tumorigenesis (3). The ubiquitin-proteasome system, a cornerstone of proteostasis, eliminates damaged proteins via E3 ligase-mediated substrate recognition (4, 5). Constitutive Photomorphogenic 1(COP1) is an E3 ubiquitin ligase. Studies have found that COP1 is abnormally expressed in various types of cancer, and it participates in the progression and metastasis of tumors by regulating the ubiquitination levels of key oncogenes and tumor suppressor genes. For example, COP1 can negatively regulate the common tumor suppressor gene p53, thereby promoting the progression of breast and ovarian cancers (6). Luo et al. discovered that in colorectal cancer, COP1 is involved in forming the CUL4B-DDB1-COP1 complex, which promotes the progression of colorectal cancer by targeting and degrading the UTX protein (7).In lung cancer, Wang et al. demonstrated that decreased COP1 leads to increased accumulation of c-Jun, which subsequently inhibits HDAC3 expression, thereby enhancing histone H3 acetylation of the PD-L1 promoter and promoting the cisplatin resistance process in lung cancer cells (8). It is currently unclear whether COP1 is involved in the regulation of RCC.

Ferroptosis is an iron-dependent form of cell death characterized by cell membrane damage caused by the accumulation of intracellular lipid peroxides, and it is closely associated with the occurrence, development, and treatment resistance of various tumors (9, 10). Acyl-CoA synthetase long-chain family member 4(ACSL4) is a key enzyme that promotes ferroptosis (11). The ACSL4-catalyzed biosynthesis of arachidonoyl-CoA contributes to the execution of ferroptosis by triggering phospholipid peroxidation (12). ACSL4 affects the progression and treatment sensitivity of certain tumors. Studies have found that ACSL4 is significantly downregulated in ferroptosis-resistant cell lines. ACSL4 knockout cells were resistant to RSL3, an inhibitor of GPX4 and an inducer of ferroptosis, while ACSL4 overexpression in ferroptosis-resistant cells (such as LNCap cells and K562 cells) significantly increased their sensitivity to ferroptosis (11, 13). In RCC, Wang et al. demonstrated that AIM2 induces phosphorylation and proteasomal degradation of FOXO3a, thereby suppressing FOXO3a-mediated transcriptional activation of ACSL4. This inhibition of ACSL4-driven ferroptosis promotes tumor progression and confers resistance to sunitinib therapy (14).

This study uncovers COP1 as a novel E3 ubiquitin ligase in RCC that targets ACSL4 for K48-linked ubiquitination and degradation, thereby suppressing ferroptosis and driving tumor progression. For the first time, we directly link dysregulated ubiquitination to ferroptosis resistance, expanding the mechanistic landscape of RCC pathogenesis. These findings not only provide critical insights into COP1’s role in tumorigenesis but also establish it as a promising therapeutic target. We further propose a rationale for combining COP1 inhibition with ferroptosis-inducing agents to enhance treatment efficacy and overcome therapeutic resistance.

## Materials and methods

### Cell culture

The RCC cell lines (786-O and ACHN) were purchased from the Cell Bank, Chinese Academy of Sciences. The 786-O cell line, derived from primary clear cell RCC (ccRCC; ~70–80% of RCC cases), represents the predominant histological subtype associated with dysregulated ubiquitination and ferroptosis resistance. The ACHN cell line, originating from metastatic RCC, was selected for its aggressive phenotype and relevance to ferroptosis modulation. Both lines are widely used in RCC mechanistic studies (15, 16). ACHN cells were cultured in DMEM (Jiangsu KeyGEN BioTECH Corp., Ltd) supplemented with 10% fetal bovine serum (TransGen Biotech), while 786-O cells were maintained in RPMI-1640 medium (Jiangsu KeyGEN BioTECH Corp., Ltd). Both cultures contained 100 U/ml penicillin, 100 μg/ml streptomycin, 5% CO_2_, and were incubated at 37°C.

### Cell transfection

Plasmids targeting COP1, ACSL4, K48, and K63 were obtained from Ribobio (Guangzhou, China). Lipofectamine 8000 (Beyotime Biotechnology, Shanghai, China) was used for cell transfection. COP1 truncation and pcDNA3.1 framework overexpression vectors were constructed from Medjaden Inc. China.

### Quantitative real-time PCR

RNA was extracted using TRIzol (Invitrogen), and the qRT-PCR process was performed using the UltraSYBR One Step RT-qPCR kit (Cwbiotech, China) and a LightCycler^®^ 96 fluorescence quantitative PCR instrument (Roche, Switzerland). Using β-actin as the internal reference, the relative gene expression was determined by the 2-ΔΔCt method. The assay was performed three times independently.

### Western blot analysis and antibodies

After separation by SDS-PAGE (P1200, Solarbio, China), the total protein was transferred to a PVDF membrane (Millipore, USA). Incubate the membrane with the primary antibody overnight at 4°C. The blot is then washed 3 times with PBS and then incubated with the secondary antibody for 1 h in a dark chamber. Specific primary antibodies against COP1 (ab56400, Abcam), ACSL4 (ab155282, Abcam), GAPDH (60004-1-AP, Proteintech), tubulin (2144, Cell Signaling Technology), Flag Tag (14793, Cell Signaling Technology), HA Tag (3724, Cell Signaling Technology) were used for western blot assays, all the western blot experiments were repeated at least three times.

### Cell proliferation, migration and invasion assays

We used the CCK-8 kit (Yeasen Biotechnology (Shanghai) Co., Ltd. China) to complete the CCK-8 assay, and used a standard instrument to measure the absorbance of each group of samples at a wavelength of 450 nm for cell viability detection. Transwell chamber (Corning Incorporated, USA) upper chamber was used to contain RCC cells. Transwell insert fragments covered with Matrigel (Corning Incorporated, USA) were used for invasion detection, while Transwell insert fragments without Matrigel membrane were used for migration detection. Cell counting was performed after crystal violet staining for 24 hours. This test was carried out independently in triplicate.

### Co-immunoprecipitation assay

The cell lysate was mixed with specific antibodies and incubated to allow the antibodies to bind to the target proteins and their interacting partners, overnight at 4°C. Magnetic beads were then added to collect the antibody-protein complexes, which were subsequently washed with IP lysis buffer to remove non-specifically bound proteins, and the target proteins and their interactors were finally eluted and analyzed. The assay was performed in triplicate independently.

### Flow cytometry and cytolipid peroxidation assays

The cell culture method and cell transfection operation are the same as before. Wash the cell suspension with PBS, add the prepared C11 BODIPY 581/591 (1mL serum-free medium + 5μL probe), and stain at 37°C for 15–20 minutes in the dark. Make the cells completely stained bright red; wash the cells with PBS to remove excess probe, add 600-800μL PBS to resuspend the stained cells. Cells were transferred to a flow tube; fluorescence data were collected on a Foresta flow cytometer (Agilent, USA). Data were analyzed using FlowJo 7.6.1 software.

### Animal work

BALB/c nude mice (6–8 weeks old, female) were obtained from Servicebio (China) and housed under standard conditions. All animal procedures were approved by the Animal Care and Use Committee of Xuzhou Medical University. For the subcutaneous tumor model, ACHN cells stably overexpressing COP1 (OE-COP1) or vector controls (1 × 10^6^ cells/mouse) were inoculated into the ventral flanks of mice (n = 6/group). Tumor dimensions (maximum diameter a and minimum diameter b) were measured weekly using digital calipers, and tumor volume was calculated as V=(a×b)^2^/2. Mice were euthanized at 28 days post-inoculation or when tumors reached 1,500 mm³, whichever occurred first. The OE-COP1 + Fer-1 group received daily intraperitoneal injections of ferrostatin-1 (Fer-1, 2 μg/mg body weight) starting from day 7, while control groups received phosphate-buffered saline (PBS). At endpoint, tumors were excised, weighed, and analyzed for histopathological features (ACSL4, Ki67) and lipid peroxidation markers.

### Statistical analysis

SPSS 20.0 software (SPSS Inc., USA) and GraphPad Prism 7 were used for data analysis. For *in vitro* experiments (e.g., CCK-8, Transwell, flow cytometry), biological replicates (n ≥ 3 independent repeats per group) were determined based on prior studies in RCC models (11). Animal group sizes (n = 6 mice/group) were calculated using G*Power 3.1 to achieve ≥80% statistical power (α = 0.05, effect size d = 1.2 derived from pilot data). Normality and variance homogeneity were verified using Shapiro-Wilk and Levene’s tests. Two-tailed Student’s t-tests (two-group comparisons) or one-way ANOVA with Tukey’s *post hoc* test (multi-group comparisons) were applied. Kaplan-Meier survival curves were analyzed via log-rank tests. Exact p-values and 95% confidence intervals (CI) are reported in figures and tables.

## Results

### COP1 is highly expressed in RCC and promotes tumor cell proliferation, migration, and invasion, correlating with poor prognosis

**Figure 1:**
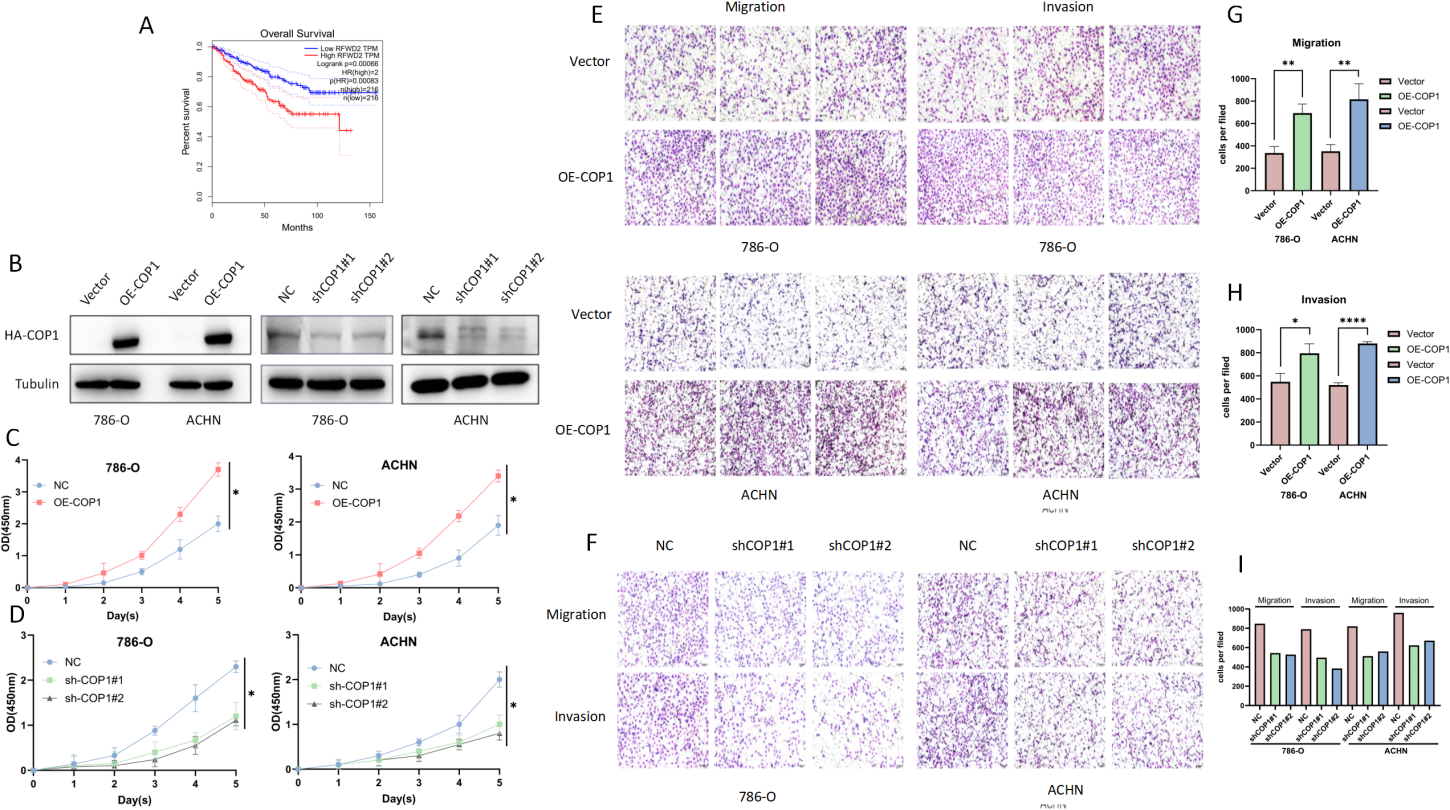
COP1 is Highly Expressed in RCC and Promotes Tumor Cell Proliferation, Migration, and Invasion, Correlating with Poor Prognosis. **(A)** Kaplan-Meier survival curves of RCC patients stratified by COP1 expression (high vs. low; log-rank test, *p <* 0.001). RFWD2 is another name for COP1. **(B)** Western blot validation of COP1 stable overexpression efficiency in 786-O and ACHN cells, and significant downregulation of COP1 after transfection with shCOP1#1/2. GAPDH served as a loading control. **(C)** CCK-8 assay demonstrated significantly enhanced cell proliferation in COP1-overexpressing 786-O and ACHN cells. **(D)** COP1 knockdown markedly suppressed cell proliferation in both cell lines compared to controls. **(E)** Transwell experiments revealed increased migratory and invasive capacities in COP1-overexpressing cells. **(F)** COP1 knockdown significantly attenuated cell migration and invasion. **(G)** The statistical analysis of the result that COP1-overexpressing cells in 786-O and ACHN RCC cells have stronger migration ability in Transwell experiments. **(H)** The statistical analysis of the result that COP1-overexpressing cells in 786-O and ACHN RCC cells have stronger invasion ability in Transwell experiments. **(I)** Statistical analysis of the result that the invasion and migration abilities of 786-O and ACHN RCC cells are downregulated after COP1 knockdown in Transwell experiments. *p < 0.05, **p < 0.01, ****p < 0.0001.

Analysis of GEPIA (Gene Expression Profiling Interactive Analysis) database (http://gepia.cancer-pku.cn/) revealed Patients with high COP1 expression exhibited shorter overall survival (**Figure 1A**). In **Figure 1A**, RFWD2 is another name for COP1. For details, please refer to https://www.ncbi.nlm.nih.gov/gene/?term=COP1. GEPIA is a web-based tool to deliver fast and customizable functionalities based on TCGA (The Cancer Genome Atlas Program) and GTEx (Genotype-Tissue Expression) data (17). We established HA-tagged COP1-overexpressing stable cell lines in 786-O and ACHN cells. Transfection with shCOP1#1/2 in these cell lines significantly downregulated COP1 expression (**Figure 1B**). Functional assays demonstrated that COP1 overexpression markedly promoted RCC cell proliferation (CCK-8 assay, **Figure 1C**), whereas COP1 knockdown suppressed proliferation (**Figure 1D**). Similarly, Transwell experiments showed that COP1 overexpression enhanced cell migration and invasion (**Figure 1E**), while its knockdown inhibited these malignant phenotypes (**Figure 1F**). The statistical analysis results of the Transwell experiments are shown in **Figures 1G–I**.

### COP1 interacts with ACSL4 and downregulates ACSL4 protein levels in RCC

**Figure 2:**
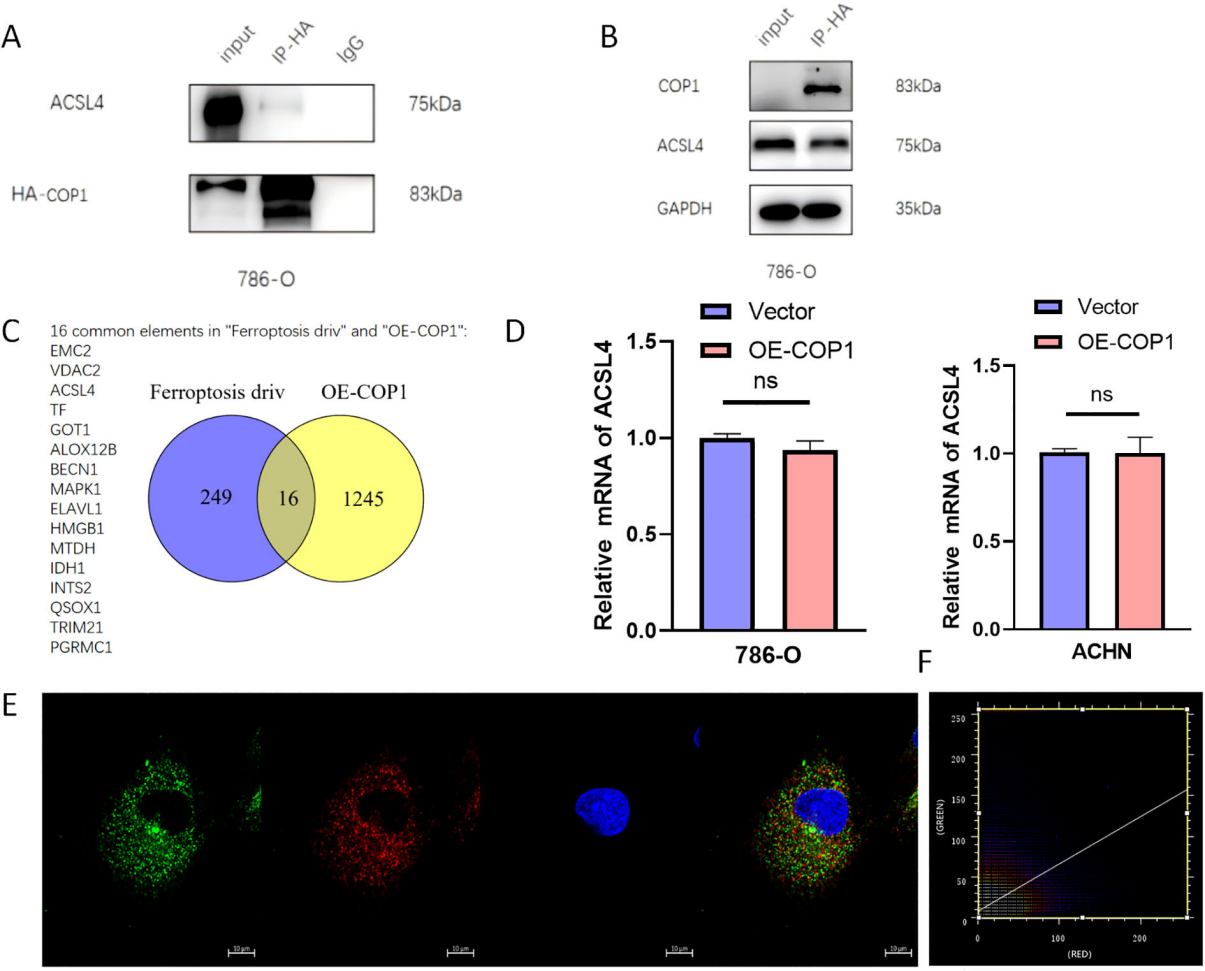
COP1 Interacts with ACSL4 and Downregulates ACSL4 Protein Levels in RCC. **(A)** CO-IP experiments in 786-O cells demonstrated a physical interaction between ACSL4 and COP1. **(B)** Western blot analysis in 786-O cells revealed that overexpression of COP1 significantly reduced ACSL4 protein levels. **(C)** Screening of the ferroptosis database identified 16 ferroptosis-related proteins, including ACSL4, that interact with COP1. **(D)** RT-PCR experiments showed no significant changes in ACSL4 mRNA levels upon COP1 overexpression in 786-O and ACHN cells. **(E)** Immunofluorescence co-localization experiments in 786-O cells confirmed the interaction between ACSL4 and COP1. The protein labeled with green fluorescence is COP1, and the protein labeled with red fluorescence is ACSL4. **(F)** Co-localization analysis of the immunofluorescence image by using ImageJ software. Pearson’s r = 0.518. ns, no significant difference in statistics.

To identify the target proteins through which COP1 exerts its effects in RCC cells, we overexpressed COP1 in 786-O cells and detected binding partners via mass spectrometry and co-immunoprecipitation (Co-IP), confirming ACSL4 as a COP1 interactor (**Figure 2A**). ACSL4 was prioritized due to its established role as a key ferroptosis regulator, catalyzing polyunsaturated fatty acid (PUFA)-phospholipid biosynthesis critical for lipid peroxidation (18, 19). Western blot analysis revealed that COP1 overexpression significantly reduced ACSL4 protein levels (**Figure 2B**), a finding corroborated by protein-binding mass spectrometry (**Supplementary Figure S1**). Screening the ferroptosis database (http://www.zhounan.org/ferrdb/current/) identified 16 ferroptosis-related proteins, including ACSL4, among 1261 COP1 interactors (**Figure 2C**). Immunofluorescence co-localization further validated the ACSL4-COP1 interaction (**Figure 2E**). Notably, COP1 overexpression did not alter ACSL4 mRNA levels (**Figure 2D**), prompting the hypothesis that COP1 promotes ubiquitination-dependent ACSL4 degradation. Co-localization analysis of the immunofluorescence image by using ImageJ software (**Figure 2F**). Pearson’s r = 0.518. Subsequent validation confirmed COP1-mediated K48-linked ubiquitination and proteasomal degradation of ACSL4, directly inhibiting ferroptosis. This mechanism aligns with prior studies implicating ACSL4 as a ferroptosis driver in cancer, underscoring its therapeutic relevance in RCC (13, 20).

### COP1 promotes ubiquitination-dependent degradation of ACSL4 and suppresses ferroptosis in RCC cells

**Figure 3:**
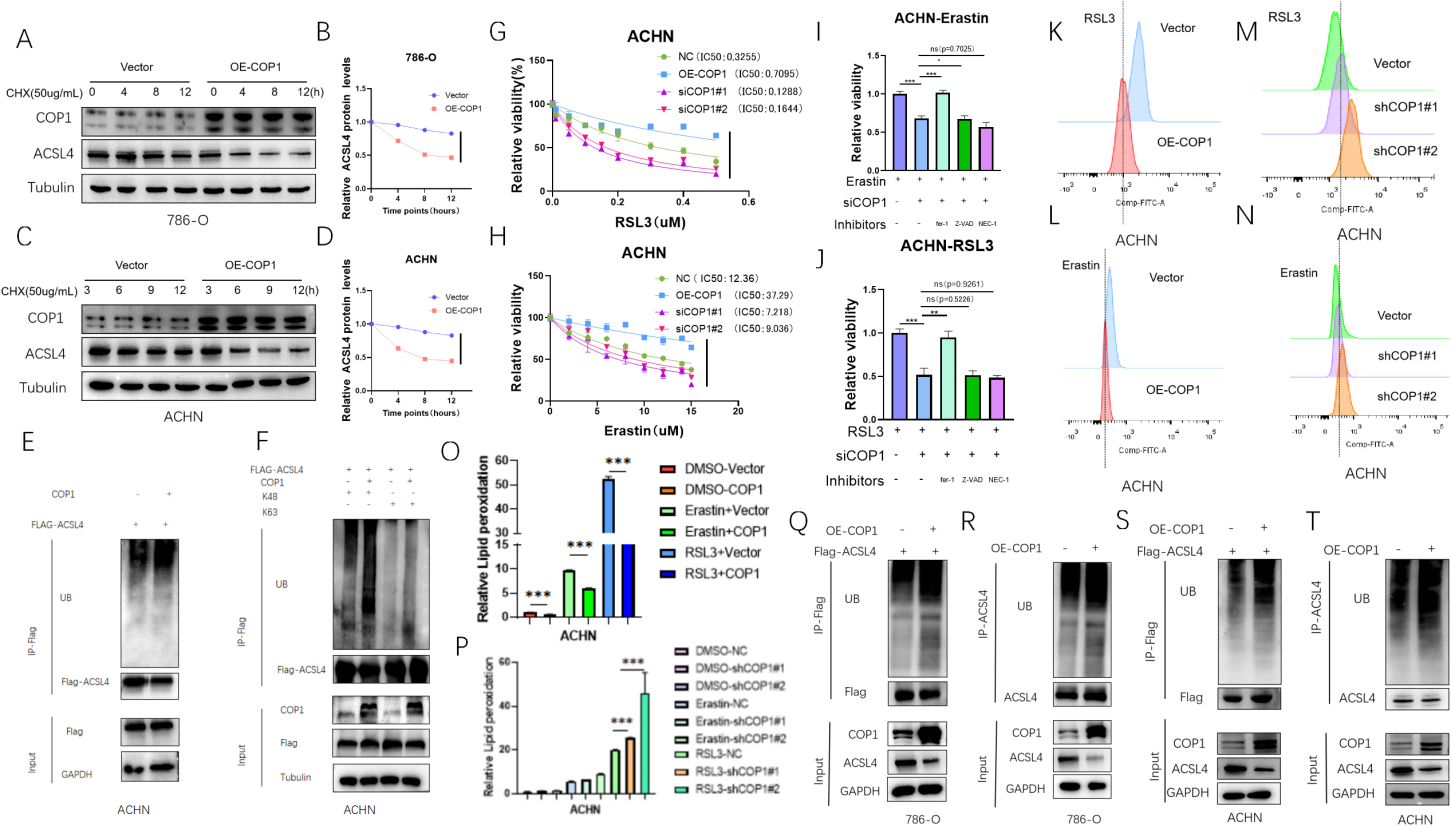
COP1 Promotes Ubiquitination-Dependent Degradation of ACSL4 and Suppresses Ferroptosis in RCC Cells. **(A)** ACSL4 and COP1 protein levels in 786-O cells (Control vs. OE-COP1) after CHX treatment (50 μg/mL). Tubulin: loading control. **(B)** Quantified ACSL4 half-life in 786-O cells (Control vs. OE-COP1). **(C)** ACSL4 and COP1 protein levels in ACHN cells (Control vs. OE-COP1) after CHX treatment. Tubulin: loading control. **(D)** Quantified ACSL4 half-life in ACHN cells (Control vs. OE-COP1). **(E)** Co-IP assays confirmed COP1-mediated ubiquitination-dependent degradation of ACSL4 in ACHN cells. **(F)** Co-IP analysis revealed enhanced K48-linked polyubiquitination of ACSL4 in ACHN cells co-transfected with COP1. **(G, H)** Cell viability assays using ferroptosis inducers (RSL3 and Erastin) showed that COP1 overexpression increased cell survival and suppressed ferroptosis, whereas COP1 knockdown reduced viability and accelerated ferroptosis. **(I, J)** Rescue experiments: Ferrostatin-1, Z-VAD-FMK, and Necrostatin-1 were applied to COP1-overexpressing ACHN cells treated with Erastin/RSL3. Cell viability was measured to validate ferroptosis specificity. **(K-P)** Flow cytometry analysis demonstrated altered lipid peroxidation levels in COP1-overexpressing or knockdown ACHN cells under RSL3/Erastin treatment. **(Q, R)** Exogenous ubiquitination assay in 786-O cells. Increased ubiquitination of ACSL4 observed in COP1-overexpressing (OE-COP1) cells compared to control (Vector) cells. **(S, T)** Exogenous ubiquitination assay in ACHN cells. Enhanced ubiquitination of ACSL4 detected in COP1-overexpressing (OE-COP1) cells relative to control (Vector) cells. *p < 0.05, **p < 0.01, ***p < 0.001.

We treated two RCC cell lines with the protein synthesis inhibitor cycloheximide (CHX) for varying durations after COP1 overexpression and assessed the half-life of ACSL4. The results showed that COP1 overexpression shortened the half-life of ACSL4, indicating that COP1 regulates ACSL4 expression by modulating its protein stability (**Figures 3A–D**). To investigate the molecular mechanism underlying COP1-mediated regulation of ACSL4, we co-transfected COP1 and ACSL4 in ACHN RCC cells. Co-expression of COP1 significantly increased the ubiquitination level of ACSL4 (**Figure 3E**). Further experiments using ubiquitination mutants (K48 and K63 ubiquitination sites) revealed that COP1 enhances ACSL4 ubiquitination specifically through the K48-linked polyubiquitination pathway (**Figure 3F**).

In ACHN cells treated with ferroptosis inducers RSL3 and Erastin, cell viability assays demonstrated that COP1 overexpression increased cell survival and suppressed ferroptosis, whereas COP1 knockdown reduced cell viability and accelerated ferroptosis (**Figures 3G, H**). To validate the ferroptosis-specific effect, we treated COP1-overexpressing or knockdown ACHN cells with the ferroptosis inhibitor ferrostatin-1 (Fer-1), apoptosis inhibitor Z-VAD-FMK, or necroptosis inhibitor Necrostatin-1. The results showed that the inhibitory effect of COP1 overexpression on ferroptosis was rescued by Fer-1 but not by Z-VAD-FMK or Necrostatin-1 (**Figures 3I, J**).

Flow cytometry analysis using the lipid peroxidation probe C11-BODIPY 581/591 revealed that COP1 overexpression significantly reduced ROS levels in ACHN cells treated with Erastin (10 μM) or RSL3 (0.5 μM) for 6 hours, further confirming its role in suppressing ferroptosis. Conversely, COP1 knockdown elevated ROS levels, promoting ferroptosis (**Figures 3K–P**).

Finally, co-expression of COP1 and exogenous ACSL4 in 786-O and ACHN cells demonstrated that COP1 overexpression enhances ubiquitination of exogenous ACSL4 (**Figures 3Q–T**). These findings collectively establish that COP1 inhibits ferroptosis in RCC cells by promoting K48-linked ubiquitination and degradation of ACSL4.

### COP1 suppresses ferroptosis and promotes tumor growth *in vivo*

**Figure 4:**
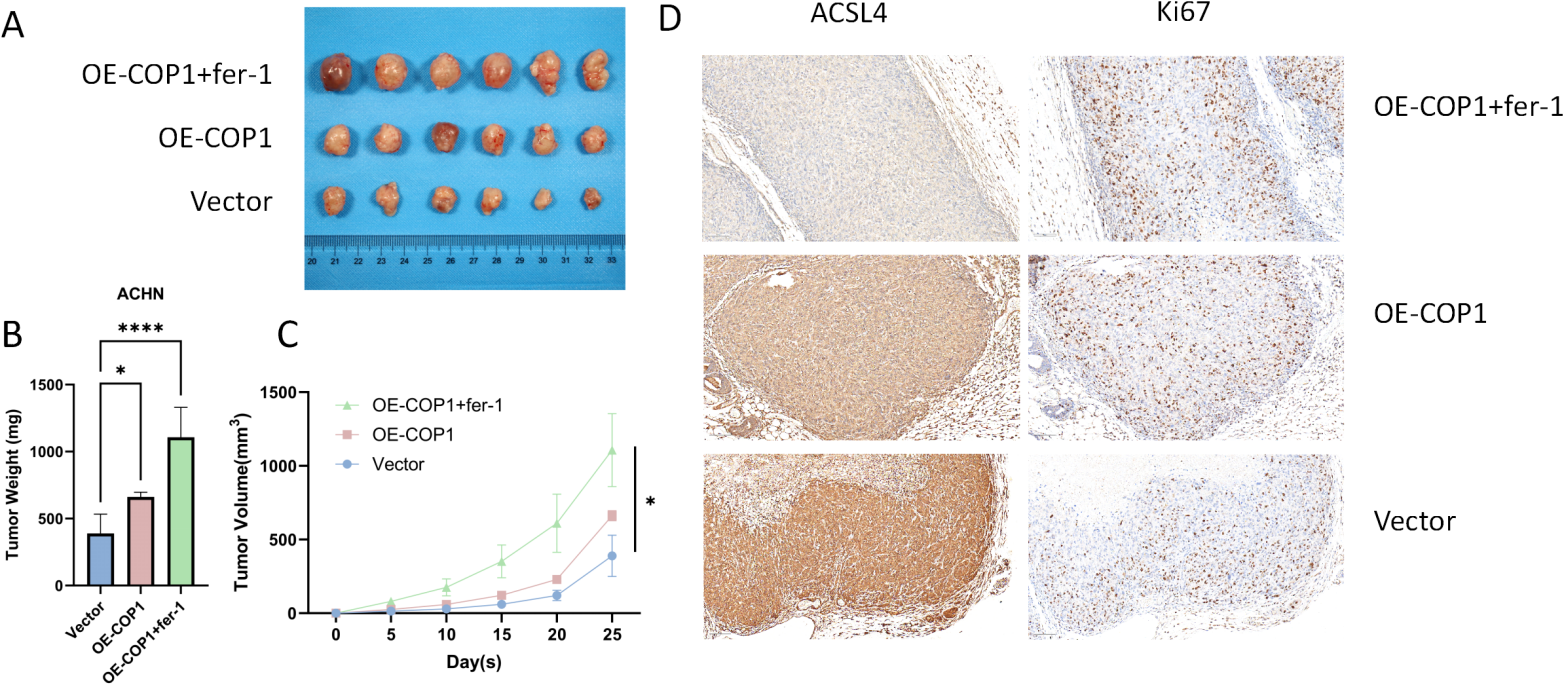
COP1 Suppresses Ferroptosis and Promotes Tumor Growth *In Vivo*. **(A)** Representative images of subcutaneous xenograft tumors in nude mice derived from ACHN cells under different treatment conditions. Six representative tumors are shown for each group. **(B, C)** Quantitative analysis of tumor weight and volume. **(D)** Representative immunohistochemical (IHC) staining images of PRMT9, GPX4, and Ki-67 in tumor sections. Scale bar, 50 μm. *p < 0.05, ****p < 0.0001.

To validate the role of COP1 in inhibiting ferroptosis in RCC cells *in vivo*, we performed a xenograft tumor model using nude mice. ACHN cells overexpressing COP1 (OE-COP1) were subcutaneously injected to establish tumors. In the OE-COP1 + Fer-1 group, mice received daily intraperitoneal injections of the ferroptosis inhibitor ferrostatin-1 (Fer-1, 2 μg/mg). Compared to the vector control group, tumors derived from COP1-overexpressing cells exhibited significantly increased tumor weight and volume (**Figures 4A–C**). Notably, Fer-1 treatment further enhanced tumor growth in the OE-COP1 group (**Figures 4A–C**). Immunohistochemical analysis revealed that COP1-high tumors displayed reduced ACSL4 expression and increased Ki67-positive proliferative cells. These effects were further amplified in the OE-COP1 + Fer-1 group, showing even lower ACSL4 levels and higher Ki67 positivity (**Figure 4D**). Collectively, these findings demonstrate that COP1 suppresses ferroptosis and drives tumor progression in RCC *in vivo*, with ACSL4 downregulation and enhanced proliferation serving as critical mechanisms.

## Discussion

As one of the core mechanisms of post-translational regulation, ubiquitination plays a pivotal role in the initiation and progression of malignant tumors. Through a cascade reaction mediated by E1-activating enzymes, E2-conjugating enzymes, and E3 ubiquitin ligases, ubiquitination precisely regulates the stability, localization, and function of target proteins, thereby influencing critical biological processes such as the cell cycle, apoptosis, DNA damage repair, and metabolic reprogramming (3). In cancer, dysregulation of the ubiquitination system frequently leads to abnormal stabilization of oncoproteins (e.g., c-Myc, Cyclin D1) or excessive degradation of tumor suppressors (e.g., p53, PTEN) (21, 22). For instance, the E3 ligase MDM2 mediates ubiquitination-dependent degradation of p53, a key mechanism underlying chemoresistance in multiple cancers (23), while the deubiquitinase USP7 stabilizes PD-L1 to promote immune evasion (24). COP1, an E3 ubiquitin ligase, has been implicated in regulating tumor cell proliferation, apoptosis, and DNA repair. Its substrates include p53, c-Jun, ETS, β-catenin, STAT3, MTA1, p27, 14-3-3σ, and C/EBPα (25). Notably, COP1 exhibits context-dependent roles in tumorigenesis, acting as either a tumor suppressor or promoter depending on its interacting targets. For example, COP1-mediated regulation of tumor suppressor p53 versus oncoprotein JUN leads to divergent outcomes in cancer progression (26, 27). However, the regulatory role of COP1 in RCC remains underexplored. In this study, we demonstrated that COP1 is overexpressed in RCC, where it promotes cancer cell migration and invasion and correlates with poor patient prognosis. Mechanistically, we revealed that COP1 directly interacts with ACSL4 and facilitates its ubiquitination-dependent degradation, providing novel insights into COP1-driven oncogenic pathways in RCC.

ACSL4, a key enzyme in lipid metabolism, activates long-chain polyunsaturated fatty acids (PUFAs, e.g., arachidonic acid) to generate acyl-CoA derivatives, thereby participating in membrane phospholipid synthesis and lipid signaling. Dysregulation of ACSL4 is associated with multiple diseases, including cancer, neurodegenerative disorders, cardiovascular diseases, acute kidney injury, and metabolic syndromes (28). Recent studies reveal that ACSL4 exhibits a dual role in cancer: while its lipid metabolic reprogramming promotes tumor progression, ACSL4 also exerts tumor-suppressive effects by driving ferroptosis, with its expression levels closely linked to cancer type, microenvironment, and therapeutic response (29). For instance, in breast cancer, ACSL4 modulates ferroptosis to influence disease progression and treatment outcomes (30). In chronic injury-dependent hepatocellular carcinoma (HCC), ACSL4 deficiency alleviates hepatocyte death and proliferation, thereby suppressing HCC development by mitigating liver injury and fibrosis (31). However, the mechanistic role of ACSL4 in RCC remains elusive. Our study uncovered and validated ACSL4-mediated ferroptosis regulation in RCC cells, demonstrating that this process is targeted via K48 ubiquitination.

Our study establishes the COP1-ACSL4 axis as a critical driver of ferroptosis suppression and malignant progression in RCC, with COP1 overexpression correlating with poor patient prognosis. While preliminary TCGA-KIRC analyses suggest genomic amplification of the COP1 locus in 12% of cases and hypoxia-driven transcriptional activation via HIF-1α, its upstream regulation may also involve post-translational modifications (e.g., AKT/mTOR-mediated phosphorylation, USP7-dependent deubiquitination) and epigenetic mechanisms (e.g., promoter hypomethylation) (8). Additionally, whether COP1 targets other ferroptosis regulators, such as GPX4 or SLC7A11, remains unresolved.

Targeting COP1 represents a promising therapeutic strategy for RCC. Small-molecule inhibitors disrupting COP1’s E3 ligase activity or substrate interactions could leverage advancements in ubiquitination-targeted therapies (32), while combining COP1 inhibition with ferroptosis inducers (e.g., erastin, RSL3) may restore ACSL4-dependent ferroptosis and overcome therapeutic resistance. Patient stratification based on COP1 expression levels, associated with aggressive disease features, could optimize therapy selection, though preclinical validation in patient-derived models and clinical trials are required to refine these approaches. These efforts will clarify COP1’s broader substrate repertoire and establish a robust foundation for precision therapies in RCC.

While our subcutaneous xenograft models confirmed COP1’s role in primary tumor growth and ferroptosis suppression, they do not fully address its contribution to metastasis. Future studies will employ advanced models—such as tail vein injection or orthotopic implantation of COP1-overexpressing RCC cells—to quantitatively assess lung metastasis formation and local invasion, aligning with its pro-migratory/invasive effects *in vitro* (33). Concurrently, we are validating COP1’s prognostic utility in multi-center cohorts and tissue microarrays (TMAs) to assess its association with metastasis and therapy resistance. Therapeutically, targeting COP1 holds promise through small-molecule inhibitors, PROTAC-based degradation, or combination therapies with ferroptosis inducers (e.g., erastin, RSL3) to restore ACSL4 and overcome resistance. HIF-2α inhibitors (e.g., belzutifan) may indirectly mitigate COP1-driven pathways, given its hypoxia-linked regulation.

To further validate the mechanistic link between COP1 and ferroptosis, ongoing efforts integrate CRISPR/Cas9 screening, ChIP-seq, and *in vivo* validation of ferroptosis markers (e.g., lipid peroxidation via C11-BODIPY, GPX4 activity). Transmission electron microscopy (TEM), a gold-standard method for identifying ultrastructural hallmarks of ferroptosis (34), will also be employed in follow-up studies to assess mitochondrial changes in COP1-overexpressing cells. Although technical constraints precluded TEM analysis in the current study, these experiments will provide complementary morphological evidence to strengthen our findings.

Several limitations warrant acknowledgment. First, while our findings are validated in preclinical models and TCGA-KIRC datasets, further confirmation in larger, multi-center clinical cohorts is needed to establish COP1’s prognostic relevance across RCC subtypes. Second, potential off-target effects of COP1 manipulation (e.g., unintended degradation of substrates or crosstalk with parallel pathways) remain uncharacterized. Proteomic profiling and CRISPR-Cas9 screens will clarify COP1’s substrate repertoire and mitigate these risks. Third, translational feasibility—optimizing inhibitor specificity or minimizing toxicity—requires rigorous preclinical validation in patient-derived organoids or xenografts. Addressing these limitations will refine COP1-directed therapeutic strategies and enhance clinical applicability. These approaches aim to advance precision therapies targeting the COP1-ACSL4 axis, improving outcomes by restoring ferroptosis sensitivity and inhibiting metastasis.

## Conclusion

**Figure 5:**
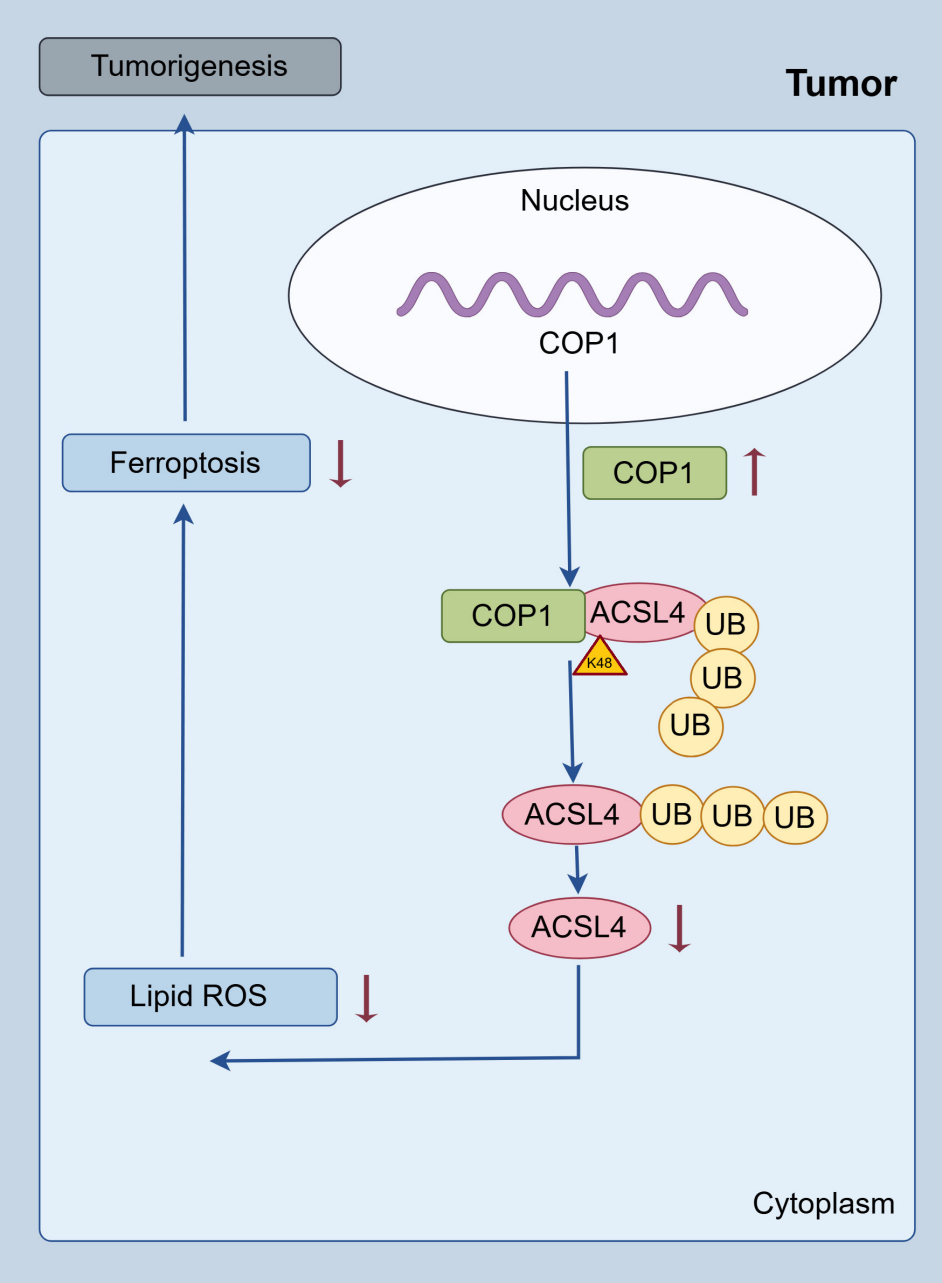
Schematic representation. Schematic representation of the proposed mechanisms.

In summary, this study elucidates a novel pathogenic mechanism in RCC by demonstrating that the E3 ubiquitin ligase COP1 directly interacts with ACSL4 and promotes its K48-linked ubiquitination and proteasomal degradation. This COP1-mediated suppression of ACSL4 protein levels significantly inhibits lipid peroxidation—a hallmark of ferroptosis—thereby conferring ferroptosis resistance to RCC cells. Functionally, this axis drives tumor progression by enhancing proliferation, migration, and invasion, while clinically, high COP1 expression correlates with aggressive disease features and poorer patient prognosis (**Figure 5**).

The findings establish the COP1/ACSL4 regulatory axis as a critical vulnerability in RCC pathogenesis. To our knowledge, this is the first study to link COP1’s E3 ligase activity to ferroptosis evasion in cancer, providing a mechanistic bridge between ubiquitination pathways and lipid metabolism-driven cell death. Therapeutically, this work highlights actionable strategies, including targeting COP1 to restore ACSL4-dependent ferroptosis and combining COP1 inhibitors with existing ferroptosis inducers (e.g., erastin, RSL3) to overcome therapy resistance. Future studies will explore COP1’s broader substrate repertoire and validate its utility as a prognostic biomarker in clinical cohorts. Collectively, these insights advance our understanding of RCC biology and lay the groundwork for precision therapies targeting ubiquitination-dependent ferroptosis regulation.

## Data Availability

The datasets presented in this study can be found in online repositories. The names of the repository/repositories and accession number(s) can be found in the article/**Supplementary Material**.

## Supplementary material

Supplementary Figure 1 Protein binding mass spectrometry analysis. Protein binding mass spectrometry analysis revealed that COP1 binds to ACSL4.
