# Supplementary figures and images for: COP1 drives renal cell carcinoma progression by targeting ACSL4 for ubiquitin-mediated degradation and inhibiting ferroptosis
Source: Front Oncol. 2025 May 6;15:1570727. doi: 10.3389/fonc.2025.1570727 (PMC12089050; doi:10.3389/fonc.2025.1570727)

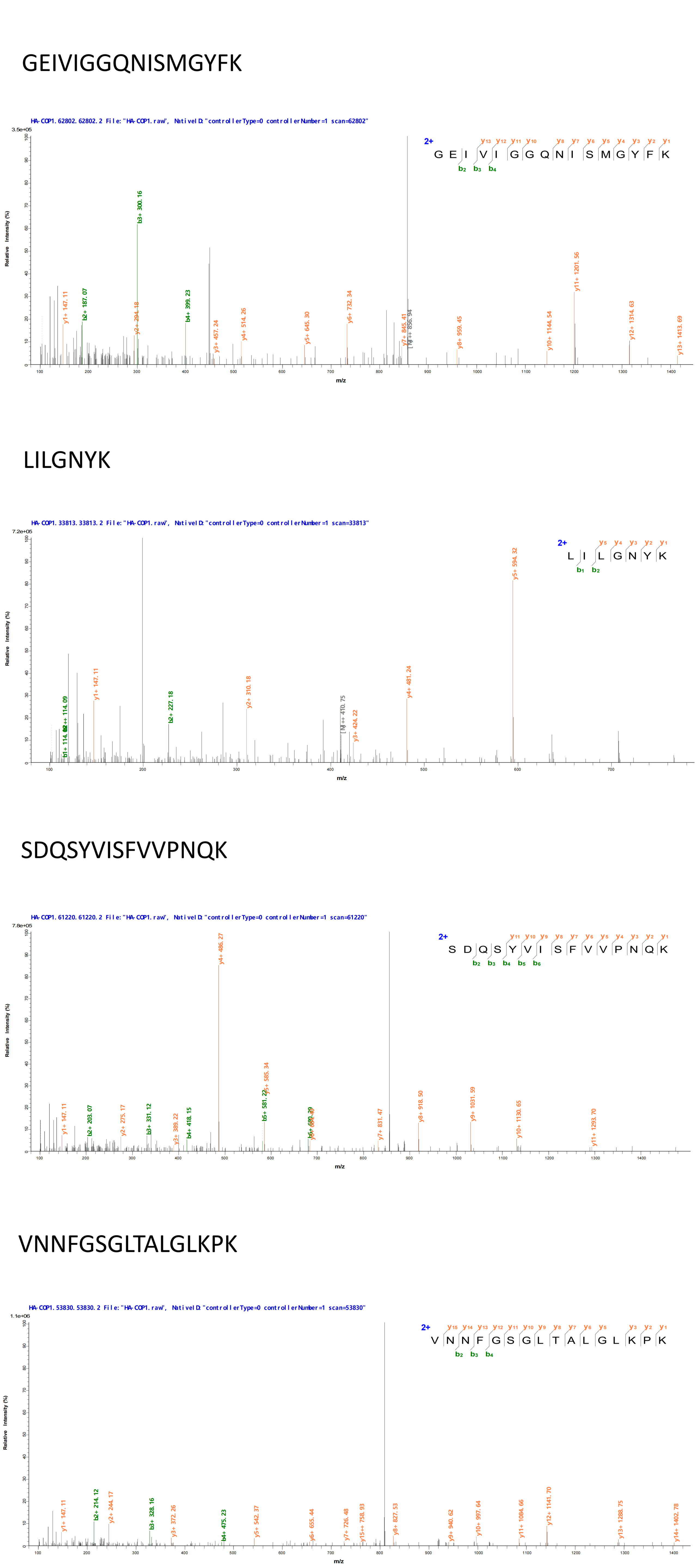

Supplement: Supplementary Figure 1 — Protein binding mass spectrometry analysis. Protein binding mass spectrometry analysis revealed that COP1 binds to ACSL4. [file Image1.tif]
